# Migrainous vertigo impairs adaptive learning as a function of uncertainty

**DOI:** 10.3389/fneur.2024.1436127

**Published:** 2024-07-25

**Authors:** Mishaal Sharif, Oliver Rea, Rose Burling, Mel Ellul Miraval, Rakesh Patel, Yougan Saman, Peter Rea, Ha-Jun Yoon, Amir Kheradmand, Qadeer Arshad

**Affiliations:** ^1^inAmind Laboratory, College of Life Sciences, University of Leicester, Leicester, United Kingdom; ^2^Faculty of Health and Life Sciences, De Monfort University, Leicester, United Kingdom; ^3^E.N.T Department, Leicester Royal Infirmary, Balance Clinic, Leicester, United Kingdom; ^4^Department of Neurology, The Johns Hopkins University School of Medicine, Baltimore, MD, United States; ^5^Department of Neuroscience, The Johns Hopkins University School of Medicine, Baltimore, MD, United States; ^6^Department of Otolaryngology and Head & Neck Surgery, The Johns Hopkins University School of Medicine, Baltimore, MD, United States; ^7^Department of Brain Sciences, Centre for Vestibular Neurology, Imperial College, London, United Kingdom

**Keywords:** dizziness, adaptive learning, risk aversion, perceptual uncertainty, vestibular migraine

## Abstract

**Objective:**

In this study, we examined whether vestibular migraine, as a source of increased perceptual uncertainty due to the associated dizziness, interferes with adaptive learning.

**Methods:**

The IOWA gambling task (IGT) was used to assess adaptive learning in both healthy controls and patients with migraine-related dizziness. Participants were presented with four decks of cards (A, B, C, and D) and requested to select a card over 100 trials. Participants received a monetary reward or a penalty with equal probability when they selected a card. Card decks A and B (high-risk decks) involved high rewards (win £100) and high penalties (lose £250), whereas C and D (low-risk decks; favorable reward-to-punishment ratio) involved lower rewards (win £50) and penalties (lose £50). Task success required participants to decide (i.e., adaptively learn) through the feedback they received that C and D were the advantageous decks.

**Results:**

The study revealed that patients with vestibular migraine selected more high-risk cards than the control group. Chronic vestibular migraine patients showed delayed improvement in task performance than those with acute presentation. Only in acute vestibular migraine patients, we observed that impaired learning positively correlated with measures of dizzy symptoms.

**Conclusion:**

The findings of this study have clinical implications for how vestibular migraine can affect behavioural adaption in patients, either directly through altered perception or indirectly by impacting cognitive processes that can result in maladaptive behavior.

## Introduction

Traditional perspectives limit vestibular functionality to gaze stabilization and maintaining spatial orientation. Emerging data challenge this perspective by illustrating bi-directional interactions linking the vestibular system to cognitive and emotional processes (1). These interactions implicate vestibular signals in domains that go far beyond those involved in the control of automatic, low-level reflexive motor circuits for balance, gaze stabilization, motion perception, and spatial orientation (1). Manifestations of these widespread interactions are also seen clinically (2). For example, following vestibular dysfunction, abnormal weighting of sensory inputs may result in high visual dependence and an increased mismatch between predicted and actual motions (3–5). This can lead to a feeling of unsteadiness or “*off-balance*” and provoke significant distress induced by heightened vigilance to both environmental factors and bodily sensations (6). In response, a significant number of patients, driven in part by their anxious temperament and personality traits, exert increased executive control over locomotion and postural dynamics (6). This is suggested to result in a maladaptive behavioral response to the demands of the acute vestibular crisis that paradoxically reduces the effectiveness of lower-level reflexive systems. Failure to disengage these maladaptive behaviors is suggested to be a key factor for transitioning from acute to chronic dizziness (6, 7).

An alternative, albeit non-mutually exclusive perspective, is that dizziness poses a challenge to the internal model that contains knowledge about the state of the body and its contextual relationship with the external world (8, 9). This model can determine the probability of an event based on established knowledge in addition to the accumulation of new evidence over time. Based on this information, behavior can be modified, allowing for adaptive learning, especially in temporally evolving environments (10, 11). In this context, if one experiences occasional, or recurrent episodes (as in chronic dizziness) random episodes that make them feel “*off-balance*,” this will not have a significant impact on behavior (i.e., low uncertainty situation). However, an unexpected, new onset of dizziness necessitates adaptive behavioral changes, for example, adjusting postural control to compensate for the new feeling of off-balance (i.e., high uncertainty situation). Consequentially, a successful adaptation would be dependent upon an individual’s ability to discriminate inconsequential variability from signals of environmental volatility that necessitate adaptive behavioral changes (12). Accordingly, here, we examined whether migraine-related dizziness, as a source of increased perceptual uncertainty, can interfere with adaptive learning in both acute and chronic patients.

## Methods

**Figure 1:**
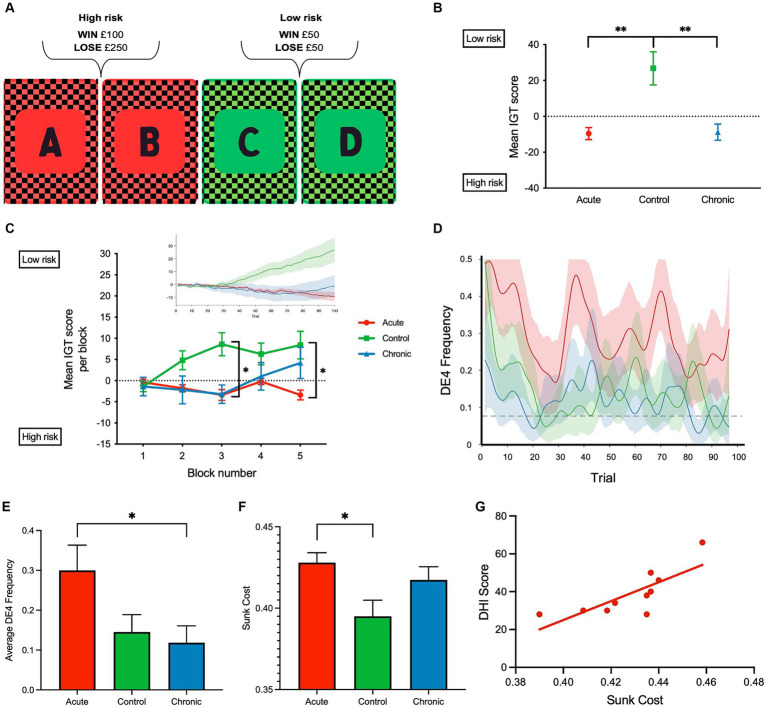
**(A)** Schematic of the computerized Iowa gambling task (IGT) implemented. **(B)** Mean IGT score for each group, respectively, was calculated for each participant across 100 trials by deducting the total number of high-risk (A, and B) card selections from the total number of low-risk card selections (C and D); Iowa gambling task (IGT) scores were calculated for each participant by deducting the total number of high-risk (A and B) card selections from the total number of low-risk card selections (C and D). A negative mean IGT score indicated that more high-risk card selections were made. **(C)** Mean IGT score per block (20 trials—5 blocks) is shown to reflect the learning rate. Insert shows the cumulative IGT score over the 100 trials. **(D)** DE4 frequency over the 100 trials and the dashed line indicates the mean empirical chance level calculated using 5,000 permutations. (**E)** Average DE4 frequency across all subject groups. **(F)** Sunk cost across all subject groups. **(G)** Correlation between dizzy symptoms (DHI) and sunk cost in aVM patients (*r* = 0.78, *p* = 0.007). **p < 0.01 and *p < 0.05. Error bars reflect SEM.

The IOWA gambling task (IGT) was used for the assessment of adaptive learning. Previous research has shown that IGT performance is linked to measures of risk assessment as well as the processing of feedback and reward as it provides insights into an individual’s ability to learn and improve performance during the task (13). Participants were presented with four decks of cards (A, B, C, and D) and requested to select a card over the course of 100 trials. Participants received a monetary reward or a penalty with equal probability when they selected a card. Card decks A and B (high-risk decks) involved high rewards (win £100) and high penalties (lose £250) with an unfavorable reward-to-punishment ratio. Card decks C and D (low-risk decks) involved lower rewards (win £50) and penalties (lose £50) with a favorable reward-to-punishment ratio (Figure 1A). Participants started with a £2,000 loan, which they were required to increase through card selection. Task success required participants to decide (i.e., adaptively learn) through feedback after each trial on their monetary gain or loss (i.e., adaptively learn) that C and D were the advantageous decks (scoring details in Figure 1). The IGT performance can additionally be measured as either sunk cost or directed exploration (DE4) index. Sunk cost reflects the expended effort in pursuit of reward, and as a maladaptive behavior, it is the tendency to continue an effort even though it is associated with higher costs than benefits (Figure 1). This was calculated by deducting the number of times an individual had a reward outcome (i.e., R or monetary reward) from the number of times with a loss outcome (i.e., L or monetary loss). Based on the risk assigned to card selections in IGT, rewards and losses from selecting high-risk cards (hR or hL) were assigned double and quadruple weights compared to low-risk cards (lR or lL). The sunk cost was then formulated as ((lL + 5^x^hL) – (lR + 2^x^hR) + 2)/7. The first term (lL + 4^x^hL) indicates the contribution of a loss outcome, while hL has a weight of “4” due to its quadruple monetary loss. Similarly, the second term (IR + 2^x^hR) indicates the contribution of a reward outcome with a double weight on hR. Theoretically, the maximum uncorrected sunk cost is 4 if a participant only chooses hL [i.e., (0 + 4×1) – (0 + 2×0) = 4], and the minimum is −2 if a participant only chooses hR [i.e., (0 + 4×0) – (0 + 2×1) = −2]. To fix the sunk cost range to be from 0 to 1, we added a correction factor of 2 and divided the result by 6. DE4 reflects the frequency at which participants selected four different decks over four consecutive trials and is a measure of randomness or uncertainty in their choices. DE4 at a time point *t* was calculated by counting the proportion of participants choosing four different decks over four consecutive trials *t + 1*, *t + 2*, and *t + 3*. We used these measures to examine the effect of recurrent dizziness on adaptive learning in relation to the duration and severity of dizzy symptoms.

Twenty right-handed patients with recurrent dizziness attributable to vestibular migraine (VM) were recruited. Previous data indicate a large (0.98) Cohen’s D for cognitive impairment in patients with vestibular dysfunction (14). Accordingly, setting alpha to 0.05 and power to 0.80 indicated that a sample of 17 patients was needed. Half of the patients had chronic vestibular migraine (cVM) (see Table 1 for demographic and further clinical details), and the other half had acute vestibular migraine (aVM). All patients conformed to the VM diagnostic criteria set out by both the Bárány Society and the Third Edition of the International Classification of Headache Disorders (15, 16). Ten matched (age, sex, and education level) healthy participants were also recruited as a control group. No participant had any other neurological, psychiatric, or otological disorder. To assess dizzy symptoms, patients completed the Dizziness Handicap Inventory (DHI), a 25-item questionnaire to assess physical, functional, and emotional factors associated with their daily dizziness (17). At the time of testing, all patients were in the interictal period and had no objective signs of vestibular dysfunction. Written informed consent was obtained from each participant (study approved by the Leicester NHS REC/IRAS 269243).

**Table 1:** Summary of demographic data and clinical characteristics.

	aVM (*n* = 10)	cVM (*n* = 10)	Control (*n* = 10)
Demographics			
Age (SEM)	44.4 (2.76)	48.1 (2.07)	40.1 (2.17)
Sex (% females)	70	70	70
Days since last symptoms (range)	11.4 (4–25)	1.8 (1–4)	–
Duration of disease in month (range)	4.1 (1–7)	15.7 (8–30)	–
Medications	0/10—prophylaxis 3/10—ibuprofen and paracetamol as needed	10/10—prophylaxis 10/10—ibuprofen and paracetamol as needed	
Clinical characteristics and questionnaire scores			
Overall DHI (SEM)	43.8 (1.95)	66.7 (1.28)	–
Functional DHI (SEM)	16.2 (0.80)	23.8 (0.56)	–
Emotional DHI (SEM)	13.2 (0.92)	20.7 (0.68)	–
Physical DHI (SEM)	14.4 (0.48)	22.2 (0.36)	–
Trait anxiety (SEM)	49.6 (4.53)	49.1 (4.97)	21.7 (2.65)
State anxiety (before experiment) (SEM)	13.9 (0.72)	16.6 (1.96)	14.4 (3.06)
State anxiety (after experiment) (SEM)	13.5 (0.69)	21 (0.93)	18.4 (1.66)

The prophylaxis medication in the cVM group was as follows: propranolol (*n* = 4), amitriptyline (*n* = 4), and topiramate (*n* = 2). State anxiety and trait anxiety were measured using the State–Trait Anxiety Inventory (STAI).

## Results

Analysis of IGT performance revealed several key findings. Both acute and chronic patient groups had lower IGT scores than the control group across the 100 trials [*F*(2, 27) = 10.19, *p* < 0.001, η*_p_*^2^ = 0.43 one-way ANOVA]. Bonferroni-corrected *post-hoc* comparisons revealed significant differences between healthy controls, aVM (*p* = 0.002) patients, and cVM (*p* = 0.002) patients, but there were no differences between aVM and cVM patients (*p* = 1.000) (Figures 1B,C). Chronic patients showed improvement in their IGT score toward the end of the task, but the acute group did not show improvement. A significant interaction was found between task blocks (block of 20 trials) and subject groups (aVM vs. cVM vs. healthy controls), showing different rates of learning among all groups [repeated measures ANOVA; *F*(2,2 7) = 7.98, *p* = 0.002, η*_p_*^2^= 0.37] (Figure 1C and insert). *Post-hoc* comparisons revealed a significant difference in the IGT score in block 3 between controls, cVM (*p* = 0.002), and aVM (*p* = 0.001) patients and between aVM patients and controls (*p* = 0.024) in block 5. Specifically, aVM patients made more high-risk selections than controls in block 5. This shows that chronic patients showed delayed learning related to risk assessment than healthy controls, while acute patients showed no improvement at all. The DE4 analysis revealed a significant difference among subject groups in their card choices [one-way ANOVA; *F*(2, 27) = 3.73, *p* = 0.03, η*_p_*^2^ = 0.22] (Figures 1D,E). Bonferroni-corrected *post-hoc* comparisons revealed differences in the DE4 between aVM and cVM, showing higher randomness in the acute patient group (*p* = 0.04). There was also a difference in sunk cost among subject groups [one-way ANOVA; *F*(2, 27) = 4.70, *p* = 0.018, η*_p_*^2^ = 0.258] (Figure 1F). Bonferroni-corrected *post-hoc* comparisons revealed a significantly higher sunk cost (i.e., cost of seeking reward) in aVM patients compared to controls (*p* = 0.018). Furthermore, measures of sunk cost were positively correlated with measures of dizzy symptoms in the aVM group (Pearson correlation *r* = 0.805; *p* = 0.005) but not in the cVM group (Pearson correlation *r* = −0.226; *p* = 0.530) (Figure 1G). This was despite significantly higher DHI scores in the cVM group compared to the aVM group (*p* = 0.01; Table 2).

**Table 2:** Summary of results and statistical tests.

		aVM	cVM	Control	*F*	*p*-value	Partial eta squared	*Post-hoc* comparisons
		Mean (SEM)						
IGT scores (100 trials)		−9.60 (3.40)	−8.80 (4.50)	26.8 (9.77)	10.191	0.0005	0.43	Controls vs. aVM: *p* = 0.002 Controls vs. cVM: *p* = 0.002 aVM vs. cVM: *p* = 1.000
Task block	Block 3	−3 0.40 (1.27)	−3.2 (2.18)	8.60 (2.73)	7.98	0.002	0.37	Controls vs. cVM: *p* = 0.002, Controls vs. aVM: *p* = 0.001
	Block 5	−3.40 (1.16)	4.20 (3.68)	8.40 (3.26)				Controls vs. aVM: *p* = 0.024
DE4 analysis		0.3 (0.06)	0.118 (0.04)	0.145 (0.04)	3.73	0.03	0.22	aVM vs. cVM: *p* = 0.04
Sunk cost		0.407 (0.007)	0.393 (0.010)	0.365 (0.012)	4.25	0.018	0.26	Control vs. aVM: *p* = 0.018

## Discussion

Taken together, the findings of this study collectively support the premise of functional interaction between vestibular perceptual dysfunction and high-level cognitive processes beyond specific triggers for symptoms (18). Both patients affected by acute and chronic dizziness displayed altered risk-based learning compared to healthy controls. Considering that dizziness can raise uncertainty due to increased perceptual noise (19, 20), its impact on adaptive learning is reflected by an inability to formulate risk-based decisions. Such observations are in keeping with previous data that reveal vestibular stimulation in healthy individuals can modulate (i) heuristics involving emotional context and framing susceptibility in risky choice games (21) and (ii) risk selection during the performance of the Balloon Analogue Risk Task (22).

An additional novel finding is that adaptive learning was impacted by the duration of symptoms. While acutely dizzy patients with vestibular migraine were impaired consistently, chronic patients showed improved scores at later IGT trials. This was observed despite more severe dizziness and higher DHI scores in the chronic group compared to the acute group. Such dissociation suggests a habituation effect with a reduced impact of dizziness on adaptive learning in chronic patients. An alternative explanation for the fact that greater dizzy symptoms in the chronic patients had less impact on task performance could potentially be linked to the fact that the higher DHI scores reported in the chronic group (>60) were unlikely to be caused solely by an organic pathology but rather reflect an increased likelihood of co-existing psychological co-morbidity [i.e., persistent postural perceptual dizziness (23)]. Further supporting the notion of a habituation effect, we observed that only acute patients showed elevated sunk cost (i.e., cost of seeking reward), which also correlated with the severity of their symptoms. This affirms the direct impact of dizziness on reward processing when there is less opportunity for habituation during the early stages of dizziness. Such an impact was dissociated in chronically dizzy patients as they showed improved adaptive learning despite more severe symptoms.

Taken together, the findings of this study have important clinical implications for how dizziness may affect cognitive functions in patients, either directly through altered perception or indirectly by impacting other cognitive or psychological processes that can result in maladaptive behavior.

## Data availability statement

The original contributions presented in the study are included in the article/supplementary material, further inquiries can be directed to the corresponding author.

## Ethics statement

The studies involving humans were approved by the Leicester NHS research ethics committee. The studies were conducted in accordance with the local legislation and institutional requirements. The participants provided their written informed consent to participate in this study.

## Author contributions

MS: Methodology, Investigation, Writing – review & editing. OR: Investigation, Methodology, Writing – review & editing. RB: Investigation, Methodology, Writing – review & editing. ME: Investigation, Methodology, Writing – review & editing. RP: Resources, Writing – review & editing. YS: Resources, Supervision, Writing – review & editing. PR: Resources, Supervision, Writing – review & editing. H-JY: Formal analysis, Visualization, Writing – review & editing. AK: Conceptualization, Formal analysis, Funding acquisition, Resources, Supervision, Writing – original draft. QA: Conceptualization, Formal analysis, Funding acquisition, Methodology, Resources, Supervision, Writing – original draft.
