# Correction: Migrainous vertigo impairs adaptive learning as a function of uncertainty

**DOI:** 10.3389/fneur.2026.1872303

**Published:** 2026-05-28

**Authors:** Mishaal Sharif, Oliver Rea, Rose Burling, Mel Ellul Miraval, Rakesh Patel, Yougan Saman, Peter Rea, Ha-Jun Yoon, Amir Kheradmand, Qadeer Arshad

**Affiliations:** 1 inAmind Laboratory, College of Life Sciences, University of Leicester, Leicester, United Kingdom; 2 Faculty of Health and Life Sciences, De Monfort University, Leicester, United Kingdom; 3 E.N.T Department, Leicester Royal Infirmary, Balance Clinic, Leicester, United Kingdom; 4 Department of Neurology, The Johns Hopkins University School of Medicine, Baltimore, MD, United States; 5 Department of Neuroscience, The Johns Hopkins University School of Medicine, Baltimore, MD, United States; 6 Department of Otolaryngology and Head & Neck Surgery, The Johns Hopkins University School of Medicine, Baltimore, MD, United States; 7 Department of Brain Sciences, Centre for Vestibular Neurology, Imperial College, London, United Kingdom

**Keywords:** dizziness, adaptive learning, risk aversion, perceptual uncertainty, vestibular migraine

In the published article there was a mistake in the formula we used to calculate the sunk cost. It was erroneously given as ((lL + 4xhL) – (IR + 2xhR) + 2)/6. The correct equation is ((lL + 5xhL) – (lR + 2xhR) + 2)/7, and therefore a correction has been made to the **Method** section.

“The sunk cost was then formulated as ((lL + 5xhL) – (lR + 2xhR) + 2)/7. The first term (lL + 5xhL) indicates the contribution of a loss outcome, while hL has a weight of “5” due to its quintuple monetary loss. Similarly, the second term (IR + 2xhR) indicates the contribution of a reward outcome with a double weight on hR. Theoretically, the maximum uncorrected sunk cost is 5 if a participant only chooses hL [i.e., (0 + 5 × 1) – (0 + 2 × 0) = 5], and the minimum is −2 if a participant only chooses hR [i.e., (0 + 5 × 0) – (0 + 2 × 1) = −2]. To fix the sunk cost range to be from 0 to 1, we added a correction factor of 2 and divided the result by 7.”

Using the updated formula, a correction has been made to the last four sentences in the **Results** section.

“There was also a difference in sunk cost among subject groups [one-way ANOVA; *F*_(2, 27)_ = 4.70, *p* = 0.018, $\eta_{p}^{2}$ = 0.258] (Figure 1F). Bonferroni-corrected *post-hoc* comparisons revealed a significantly higher sunk cost (i.e., cost of seeking reward) in aVM patients compared to controls (*p* = 0.018). Furthermore, measures of sunk cost were positively correlated with measures of dizzy symptoms in the aVM group (Pearson correlation *r* = 0.805; *p* = 0.005) but not in the cVM group (Pearson correlation *r* = −0.226; *p* = 0.530) (Figure 1G). This was despite significantly higher DHI scores in the cVM group compared to the aVM group (*p* = 0.01; Table 2).”

**Figure 1:**
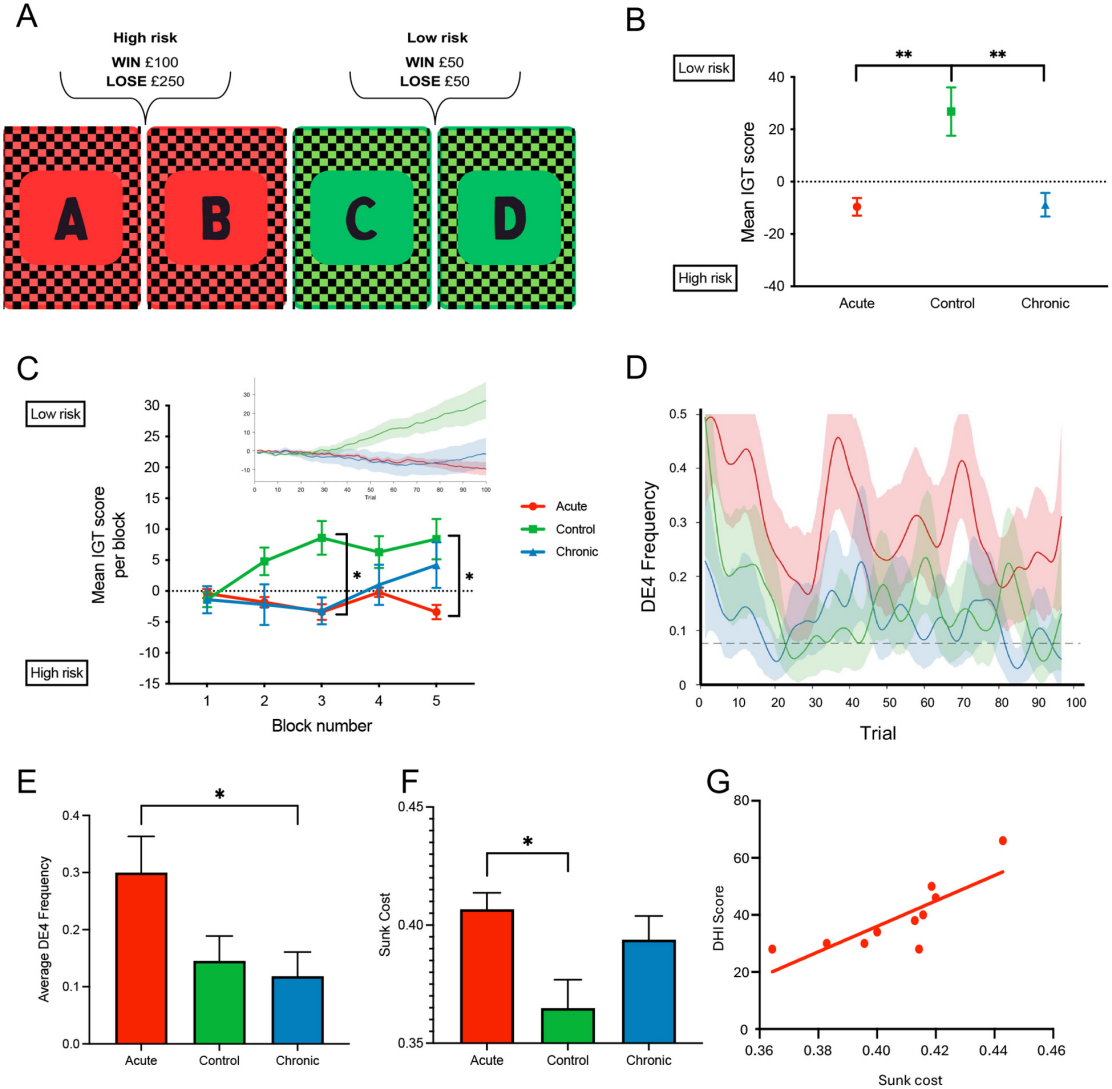
**(A)** Schematic of the computerized Iowa gambling task (IGT) implemented. **(B)** Mean IGT score for each group, respectively[[Inline Image]], was calculated for each participant across 100 trials by deducting the total number of high-risk (A and B) card selections from the total number of low-risk card selections (C and D); Iowa gambling task (IGT) scores were calculated for each participant by deducting the total number of high-risk (A and B) card selections from the total number of low-risk card selections (C and D). A negative mean IGT score indicated that more high-risk card selections were made. **(C)** Mean IGT score per block (20 trials-−5 blocks) is shown to reflect the learning rate. Insert shows the cumulative IGT score over the 100 trials. **(D)** DE4 frequency over the 100 trials and the dashed line indicates the mean empirical chance level calculated using 5,000 permutations. **(E)** Average DE4 frequency across all subject groups. **(F)** Sunk cost across all subject groups. **(G)** Correlation between dizzy symptoms (DHI) and sunk cost in aVM patients (*r* = 0.78, *p* = 0.007). ^**^p < 0.01 and ^*^p < 0.05. Error bars reflect SEM.

**Table 2:** Summary of results and statistical tests.

		aVM	cVM	Control	*F*	*p*-value	Partial eta squared	*Post-hoc* comparisons
		Mean (SEM)						
IGT scores (100 trials)		−9.60 (3.40)	−8.80 (4.50)	26.8 (9.77)	10.191	0.0005	0.43	Controls vs. aVM: *p* = 0.002 Controls vs. cVM: *p* = 0.002 aVM vs. cVM: *p* = 1.000
Task block	Block 3	3.04 (1.27)	−3.2 (2.18)	8.60 (2.73)	7.98	0.002	0.37	Controls vs. cVM: *p* = 0.002 Controls vs. aVM: *p* = 0.001
	Block 5	−3.40 (1.16)	4.20 (3.68)	8.40 (3.26)				Controls vs. aVM: *p* = 0.024
DE4 analysis		0.3 (0.06)	0.118 (0.04)	0.145 (0.04)	3.73	0.03	0.22	aVM vs. cVM: *p* = 0.04
Sunk cost		0.407 (0.007)	0.393 (0.010)	0.365 (0.012)	4.25	0.018	0.26	Controls vs. aVM: *p* = 0.018

Similarly, Table 2 and Figures 1F, G have been updated, and the corrected Table 2 and Figure 1 appear below.

The original article has been updated.

